# Bivalirudin-hydrogel coatings of polyvinyl chloride on extracorporeal membrane oxygenation for anticoagulation

**DOI:** 10.3389/fcvm.2023.1301507

**Published:** 2023-12-15

**Authors:** Wenqing Gao, Hechen Shen, Yun Chang, Qin Tang, Tong Li, Di Sun

**Affiliations:** ^1^ Department of Cardiac Center, Tianjin Third Central Hospital, Tianjin, China; ^2^ Tianjin Key Laboratory of Extracorporeal Life Support for Critical Diseases, Tianjin, China; ^3^ School of Medicine, Nankai University, Tianjin, China; ^4^ Tianjin ECMO Treatment and Training Base, Tianjin, China; ^5^ The Third Central Clinical College of Tianjin Medical University, Tianjin, China; ^6^ Department of Ophthalmology, West China Hospital Sichuan University, Chengdu, Sichuan, China; ^7^ Key Laboratory of Photochemical Conversion and Optoelectronic Material, Technical Institute of Physics and Chemistry, Chinese Academy of Sciences, Beijing, China

**Keywords:** extracorporeal membrane oxygenation, Bivalirudin, hydrogel coating, polyvinyl chloride, biocompatibility, anticoagulation

## Abstract

**Introduction:**

Thromboembolic events associated with extracorporeal membrane oxygenation (ECMO) in clinical treatment are typical. Heparin coating has been widely employed as a surface modification strategy for ECMO tubes. However, its clinical application is often accompanied by unavoidable complications due to its mechanism of action. As a direct thrombin inhibitor with a single target, Bivalirudin (BV) has exhibited a lower incidence of adverse events and superior pharmacokinetic performance compared to heparin.

**Methods:**

A gelatin methacrylate hydrogel (GelMA) coating layer with BV was successfully synthesized on polyvinyl chloride, and the drug release ratio was close to complete release within 7 days.

**Results and discussion:**

Simulated extracorporeal circulation experiments using roller pumps *in vitro* and jugular arteriovenous bypass experiments in rabbits demonstrated its outstanding anticoagulant efficacy. The systemic anticoagulant assay proved that BV hydrogel coating does not affect the coagulation level, and reduces the risk of complications such as systemic bleeding compared to intravenous injection. BV-Coating GelMA hydrogel tube has exhibited good biocompatibility and significantly improved anticoagulant performance, making it an optimal choice for surface materials used in blood-contacting medical devices.

## Introduction

As an artificial organ, ECMO could provide adequate cardiopulmonary support for critically ill patients with cardiopulmonary failure. However, there are many concurrent problems associated with treatment. Due to the direct contact between ECMO and blood, there may be an imbalance among the coagulation and anticoagulation mechanisms, ultimately leading to bleeding and thrombotic events (1). Therefore, modifying the surface of the ECMO tube with anticoagulant coatings could effectively reduce the occurrence of complex pathological and physiological reactions that arise from blood contact, thus minimizing clinical adverse events.

Heparin was the most commonly utilized coating technology in clinical practice during ECMO adjunctive therapy (2). However, due to the anionic nature of heparin molecules, heparin could bind to platelets and plasma proteins, resulting in heparin-induced thrombocytopenia (HIT) and incalculable depletion of heparin levels in the blood (3). Therefore, it is necessary to closely monitor the active clotting time during treatment to ensure the drug concentration remains within the appropriate range. BV could improve in-hospital mortality and exhibits reversible and controllable binding to thrombin during ECMO supportive compared to heparin (4). BV could directly inhibit thrombin without the need for antithrombin activation. Above all, BV could not interact with heparin-induced antibodies, avoiding the potential risk of life-threatening allergic syndrome and HIT (5). It is particularly important to effectively carry carriers on the surface of BV-loaded PVC.

Hydrogels have found extensive application in contexts intimately associated with biological organisms (6) due to the controlled three-dimensional network structure, remarkable flexibility, water solubility, and biocompatibility. By adjusting the internal structure of the hydrogel and the interactions between the molecules of the drug (such as hydrophobic interactions and covalent bonding), hydrogel polymers could effectively modulate the rate of drug release (7). Functional hydrogel coatings play a significant role in the field of medical devices, e.g.: blood-contacting (8), needles (9), dental (10), catheters (11), et al. GelMA has gained widespread application in tissue engineering (12) due to its biological properties and tunable properties to the synthesized hydrogel.

**Figure 1:**
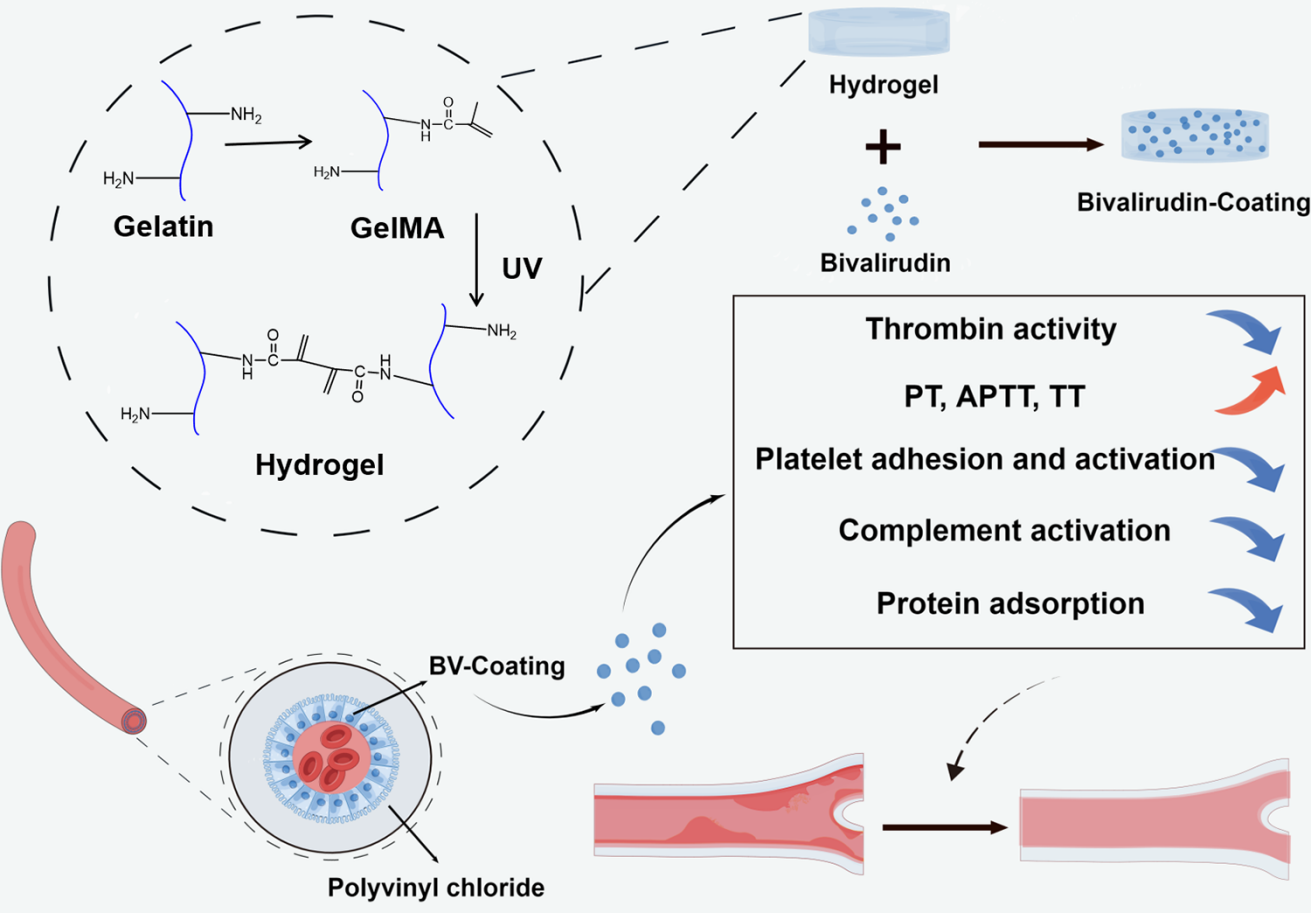
Schematic illustration of the synthesis of GelMA hydrogel coating layer with BV and the anticoagulant effect of ECMO tube.

Sodium alginate-loaded hydrogel coatings on polyvinyl chloride (PVC) tube has developed and confirmed the efficacy of the anticoagulant tube of ECMO (13). Meanwhile, an integrated hydrogel tube with a hollow core-shell-shell structure was synthesized to fulfill the anticoagulant criteria for the inner tube layer and provide the required highly elastic soft material for the outer layer (14). Here, a GelMA hydrogel coating layer with BV was synthesized on a PVC tube, and BV-loaded GelMA hydrogel coating has potential application in the anticoagulation of medical device pipeline (Figure 1).

## Materials

Gelatin (Type A, 300 bloom from porcine skin), methacrylic anhydride (MA), and Irgacure I-2959 (≥95%) were purchased from Sigma-Aldrich (St. Louis, MO, USA). Mouse fibroblast-like (L929) cells were obtained from the American Type Culture Collection. BV, phosphate-buffered saline (PBS), bovine serum albumin (BSA), bicinchoninic acid assay (BCA) kit, cell counting kit-8 (CCK-8) kit, Human Platelet rich plasma (PRP) separation kit and paraformaldehyde were obtained from Solarbio (Beijing, China). Dulbecco's modified eagle medium (DMEM), penicillin-streptomycin, and fetal bovine serum were obtained from Priscilla (Wuhan, China). Enzyme-linked immunosorbent assay (ELISA) of human complement fragment 3a (C3a) kit was purchased from MEIMIAN (Jiangsu, China). Qisong Biotechnology (Qingdao, China) provided Human β-thromboglobulin (β-TG) assay kit. Calcein/PI cell viability/cytotoxicity assay kit and sodium dodecyl sulfate (SDS) were obtained from Beyotime (Shanghai, China). The kits of clotting factors II, V, and X were purchased from the Jihe Biotechnology (Shanghai, China).

## Methods

### Synthesis of GelMA and BV-based GelMA hydrogel

Briefly, 5 g gelatin in PBS was heated to 50 °C and stirred until complete dissolution. Methacrylic anhydride was subsequently added to continue for 4 h. Subsequently, the mixture underwent dialysis against distilled water for 3 days by dialysis with a cutoff of 12–14 kDa to eliminate any residual methacrylic anhydride. Finally, the solution was lyophilized to yield thoroughly dried GelMA (15).

We optimized the coating to clean using an ammonia plasma, washed with ethanol, and then completely dried to increase surface hydrophilicity (13). 20 wt% GelMA, 1 wt% Irgacure I-2959, and 0 or 5 mg/ml BV in water completely flow through the inner cavity of the pipeline, then were exposed to ultraviolet ray (WFH-204B, λ = 365 nm, 12 W, hang zhou qi wei instrument co.ltd, China) for 30 min to create the interpenetrating hydrogel layer on the PVC surface.

### ^1^H nuclear magnetic resonance (^1^H NMR) spectroscopy

^1^H NMR spectroscopy was performed on GelMA. 1 wt% Gelatin and GelMA were dissolved in deuterium oxide (D_2_O) and subjected to analysis via a 500 MHz Fourier Transform Nuclear Magnetic Resonance (FT-NMR) spectrometer (Varian, Palo Alto, CA, USA) and software tools within the Mestrenova.

### Drug release

The drug release efficiency of BV was quantitatively determined using the BCA kit. A standard curve was constructed by diluting BSA at different concentrations. A BV-loaded GelMA hydrogel tube was placed in 10 ml of PBS and incubated for 12 days. 200 µl of supernatant was collected and co-incubated with the BCA reagent to validate the daily drug release trend. The concentration was measured at a wavelength of 562 nm using the BCA protein quantification kit and a microplate reader instrument (Tecan, Infinite F50, Switzerland). The release rate of BV was calculated using the following formula (1):

(1) $$\mathrm{Release}\;\mathrm{rate}\;(\text{\%})=\frac{\mathrm{Cumulative}\;\mathrm{release}}{\mathrm{Actual}\;\mathrm{load}}\times 100\text{\%}$$

Finally, to evaluate the release behavior of BV in GelMA hydrogel, the zero-order release model, first-order release model, Higuchi release model, and Ritger-Peppas release model were calculated to fit the release curve (16).

### Hemolysis rate

The hemolysis rate could reflect the extent to which a substance causes red blood cells (RBC) to rupture after contact with blood (17). The pristine PVC, GelMA hydrogel, and BV-loaded GelMA hydrogel tube were divided into the same size segment (1 cm). All segments were incubated separately in DMEM (1 ml) at 37 °C to obtain the extracted solution (18).

Experimental groups were established, including a positive control group (consisting of a combination of 50 µl of RBC and 950 µl of pure water), a negative regular group (consisting of 50 µl of RBC and 950 µl of PBS), and a sample group (comprising 500 µl of the extracted solution, 50 µl of RBC, and 450 µl of PBS). All groups were subjected to an incubation period of 1.5 h at 37 °C, followed by centrifugation at 2,500 rpm for 5 min. Subsequently, the absorbance of the sample was measured using a microplate reader instrument at a wavelength of 540 nm.

The hemolysis rate was calculated using the following formula (2):

(2) $$\mathrm{Hemolysis}\;\mathrm{ratio}\;(\text{\%})=\frac{A_{s}-A_{n}}{A_{p}-A_{n}}\;\times \;100\text{\%}$$

As: absorbance of the test sample; An: absorbance of the RBC suspension in the negative control; Ap: absorbance of the RBC suspension in the positive control. A threshold value of absorbance less than 5% is considered acceptable for hemolysis.

### Complement and platelet activation without flowing *in vitro*

Fresh whole blood was collected from healthy adults, and PRP was isolated using a Human PRP separation kit. Different materials were incubated with PRP at 37 °C for 1 h. The β-TG content, indicative of platelet activation, was measured using a Human β-TG assay kit.

The PRP extracted above was added to the 24-well plates containing the same size (1 cm) of different materials and incubated for 1 h. Subsequently, an ELISA kit of C3a was employed to measure the concentration of C3a in the plasma. Measuring C3a levels will provide insights into the interaction between the materials and the immune.

### Cytotoxicity

The biological characteristics of the BV-Coating GelMA hydrogel tube were evaluated using L929 cells. The cells were cultivated in a DMEM supplemented with 1% penicillin-streptomycin and 10% fetal bovine serum. The culture was upheld at 37 °C in a humidified atmosphere with 5% CO_2_.

The L929 cells were incubated with the extracted solution for 48 h. CCK-8 assay kit was used to test the cytotoxicity using a microplate reader instrument. Calcein/PI cell viability/cytotoxicity was observed and captured by a fluorescent inverted microscope (BDFACSCantoTMIIF lo, Japan).

### Blood clotting time without flowing *in vitro*

The blood in the experiments was obtained from Tianjin Third Central Hospital, and the Institutional Review Board has approved the relevant protocols. Fresh whole blood was collected and centrifuged for 15 min (2,500 rpm) to obtain Platelet-Poor Plasma (PPP). The platelets were incubated with the same size (1 cm) of different materials for 30 min. After the incubation, the activated partial thromboplastin time (APTT), thrombin time (TT), and fibrinogen (FIB) and prothrombin time (PT) of the PPP were measured using automatic coagulation analyzer (Diagnostica Stago, STA-R Evolution, France).

### Platelet adhesion and protein adsorption without flowing *in vitro*

PRP was isolated using a Human PRP separation kit. Three material groups were incubated with PRP at 37 °C for 1 h. The difference in platelet counts before and after incubation was calculated using hematology analyzers (Sysmex XN-1000, Kobe, Japan) to indirectly assess the number of platelets adhering to the inner surface of the tube.

BCA assay was employed to quantify the protein adsorption of the materials. The adsorption of proteins was evaluated through co-incubation with BSA. Same size (1 cm) of different materials was incubated in 1 ml of BSA solution (1 mg/ml in PBS) for 2 h. Then, all samples were placed in a PBS solution containing 2 wt% SDS for shaking incubation. The remaining protein concentration in the SDS solution was measured using a BCA assay kit.

### Simulate the circulation *in vitro*

Three material groups with a length of 35 cm were selected to simulate the circulation of the ECMO system with a flow rate of 2.5–3.5 L/min by the centrifugal pump of the ECMO machine. After 4 h of operation, the samples of whole blood contained within the tube were poured into Petri dishes. Subsequently, the tube was gently rinsed once, and weighed, and the results were compared to its initial weight.

### Circulation *in vivo*

Three New Zealand rabbits were acquired from the Beijing Vital River Laboratory Animal Technology, and the institutional review board of Tianjin Third Central Hospital has approved the relevant protocols. The adult male rabbits (2.2–2.5 kg) were anesthetized using urethane (5 ml/kg) via the marginal ear vein. An external pristine PVC tube connected the left carotid artery and right jugular vein (18, 19). PVC tube coated with GelMA hydrogel and BV-loaded GelMA hydrogel tube was parallel to the external pristine PVC tube, simultaneously. An extracorporeal bypass was established for 4 h to observe the formation of blood clots.

After circulation, samples of tubes were photographed, fixed, and dehydrated. The surface blood clots were collected and weighed. The morphology of the samples was observed using a scanning electron microscope (SEM, Aztec Live ULTIM, ThermoFisher Scientific, USA).

### Comparison of systemic anticoagulant assay *in vivo*

Nine New Zealand rabbits circulation experiments, including pristine PVC tube, BV-Coating GelMA hydrogel tube, and PVC tube with traditional intravenous administration of BV (BV-iv, 2.5 mg/kg/h), respectively.

At 20, 40, 60, and 80 min after the start of circulation, cut 0.5 cm at different parts of each rabbit's ear auricular vein to measure the bleeding time.

Rabbit blood was collected through the auricular vein after circulation for 4 h. The depletion of coagulation factors is a key issue in the long-term use of ECMO, the relevant important clotting factors (II, V, and X) (1, 20) and blood coagulation (PT/APTT) were also tested in the whole blood of rabbits.

### Statistical analysis

All data are presented as a mean ± standard deviation. Statistical analysis was performed using a one-way ANOVA. *P-*value < 0.05 was considered statistically significant.

## Results

### Gelma synthesis and drug deliever

**Figure 2:**
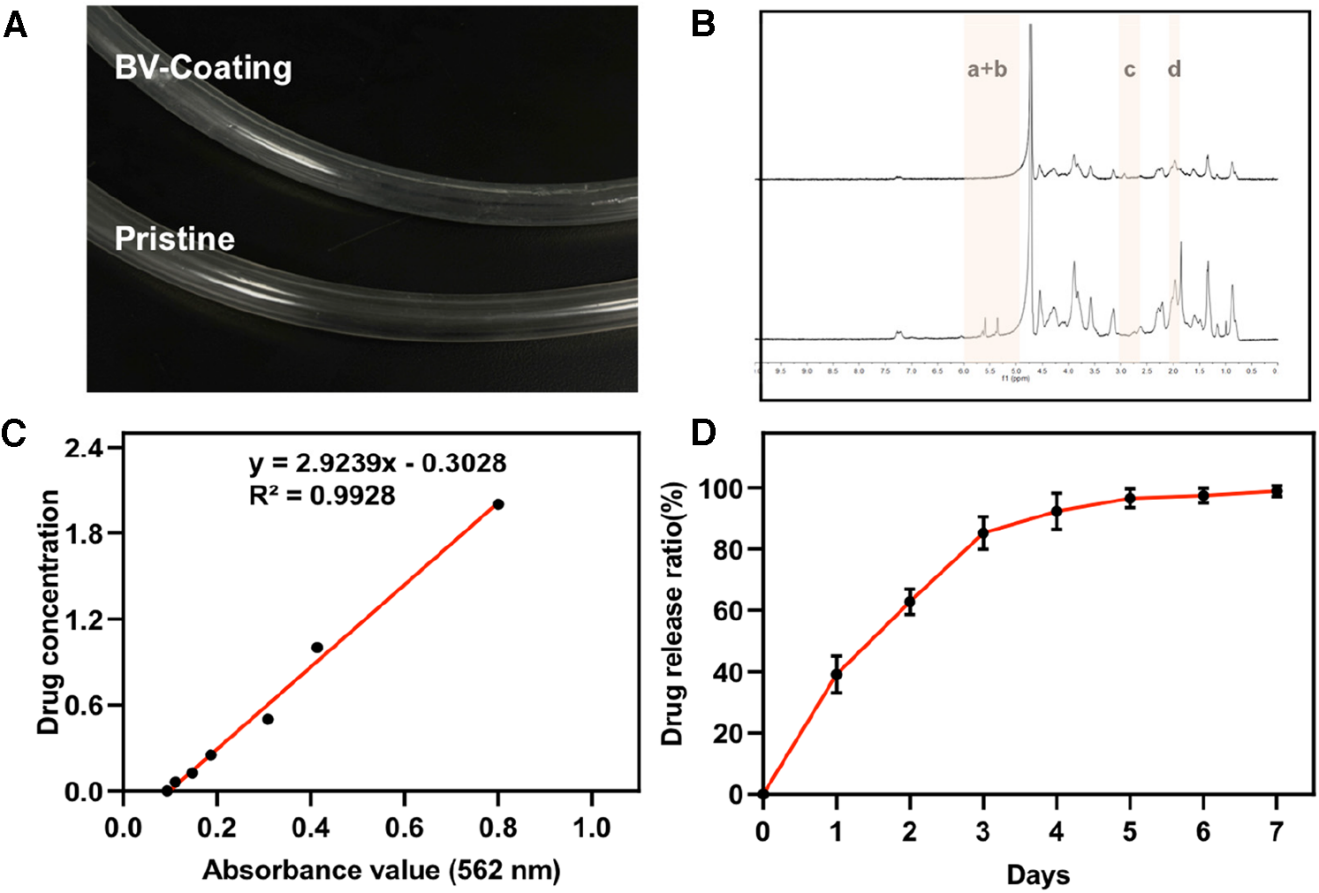
GelMA synthesis and drug release. (**A**) The image of GelMA-hydrogel coatings tube and prisitine tube. (**B**) Proton nuclear magnetic resonance (^1^H-MNR) spectra of gelatin and GelMA. (**C**) Standard curve of ultraviolet absorption of BV at 562 nm. (**D**) Performance test of drug release from BV-Coating within 7 days.

The BV-Coating GelMA hydrogel tube exhibited transparency, which facilitated enhanced observation of thromboembolism in clinical settings (Figure 2A and Supplementary Figure S1). GelMA was synthesized through the reaction of gelatin with methacrylic anhydride, which was subsequently characterized by ^1^H NMR (Figure 2B) and the details on the modification (Supplementary Figure S2). In the 5–6 ppm range (indicated as peaks a + b), the GelMA spectrum displayed discernible peaks corresponding to the acrylic protons of the methacryloyl grafts of lysine and hydroxylysine groups. Notably, peak c was significantly smaller in the GelMA spectrum at 2.9 ppm, indicating successful functionalization of a significant number of lysine groups. Furthermore, at 1.8 ppm, Peak d was discernible in the GelMA spectrum, attributed to the methyl protons of methacryloyl grafts. These ^1^H-NMR spectra conclusively confirmed the successful methacryloyl functionalization of gelatin.

The standard ultraviolet absorption curve of BV was determined by BCA (Figure 2C). The equation of the standard curve was: *Y* = 2.9239*x*−0.3028, and the correlation coefficient was 0.9928. These results show that the absorbance of BV solution has an appropriate linear relationship with the concentration range of 0.25–2.5 mg/ml concentration. Based on this, the release curve of BV among GelMA hydrogel within 7 days was measured. As was shown in Figure 2D, the rapid release period of BV was within the first 3 days. Subsequently, the release of BV was gradually equalized over 3–5 days. By comparing the adjusted R-Square sizes of the four model fitting processes, it was found that the drug release process was more consistent with the first-order release equation fitting (Supplementary Figure S3 and Table S1). Meanwhile, the Higuchi and Ritger-Peppas model could also fit the BV release process well. The drug release performance test confirmed that the release effect of BV was changed with the extension of time, and no swift drug release effect was exposed. These results showed that the BV-Coating GelMA hydrogel tube has sustained release performance under certain conditions, which could be applied to the ECMO tube to achieve the expected clinical release effect.

### Hemolysis rate

**Figure 3:**
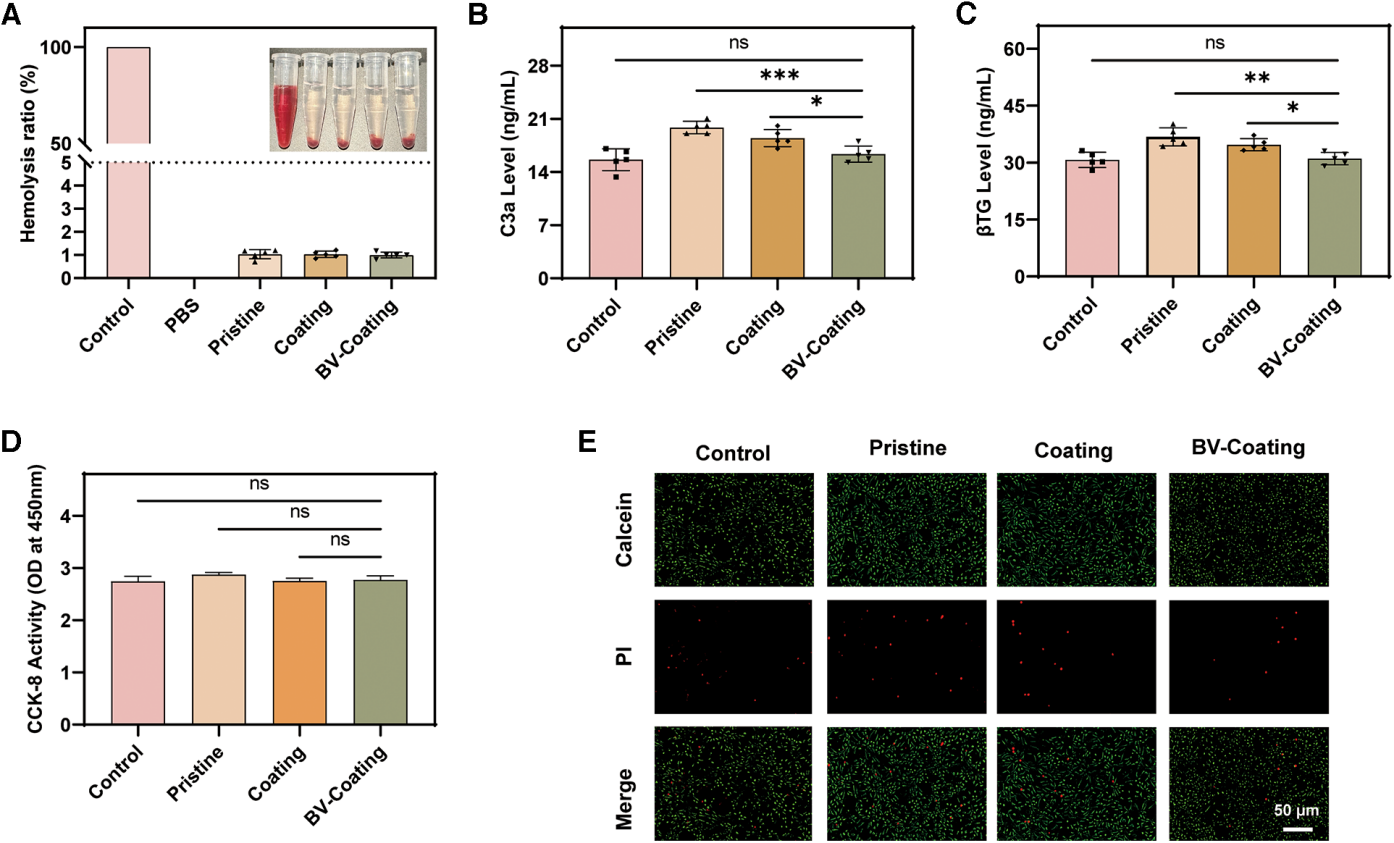
*In vitro* biocompatibility assay. (**A**) Hemolysis analysis; (**B**) Complement activation level index of C3a; (**C**) Platelet activation indicator level of β-TG; (**D**) Cytocompatibility; (**E**) Cell viability. (****P < 0.0001; ***P < 0.001; **P < 0.01; *P < 0.05; ns, not significant).

Following the criterion of ISO 10993-4.52, the hemolysis ratio should be less than 5% when the material is in direct contact with blood (21). As shown in Figure 3A, the hemolysis rates for the pristine, coating, and BV-Coating groups were recorded as 1.03%, 1.02%, and 0.99% respectively. All the results demonstrated that the hemolysis ratios were below the specified threshold of 5%, indicating that all the materials exhibit safe compatibility with blood.

### Complement and platelet activation without flowing *in vitro*

ELISA of C3a and β-TG was selected to measure the extent of complement and platelet activation platelet activation status. As shown in Figures 3B,C, the expression of C3a and β-TG in the BV-Coating group was significantly lower than that in the pristine group (*P *< 0.01). Compared to the control group, the BV-Coating group showed no statistically significant difference in the expression of C3a and β-TG, suggesting that the BV-Coating GelMA hydrogel tube could restrain complement activation and additional platelet activation.

### Cytotoxicity

The CCK-8 results (Figure 3D) demonstrated that when cultured in the extracts of different materials, cells exhibited growth trends similar to that of the control group after 48 h. Additionally, the immunofluorescence staining results (Figure 3E) showed that a large number of viable cells were stained green, and only a minimal number of dead cells were stained red after 48 h. Therefore, GelMA hydrogel coating loaded with BV could exhibit no significant cytotoxicity toward cells, suggesting it is an appropriate biocompatible biomaterial.

### Blood clotting time without flowing *in vitro*

**Figure 4:**
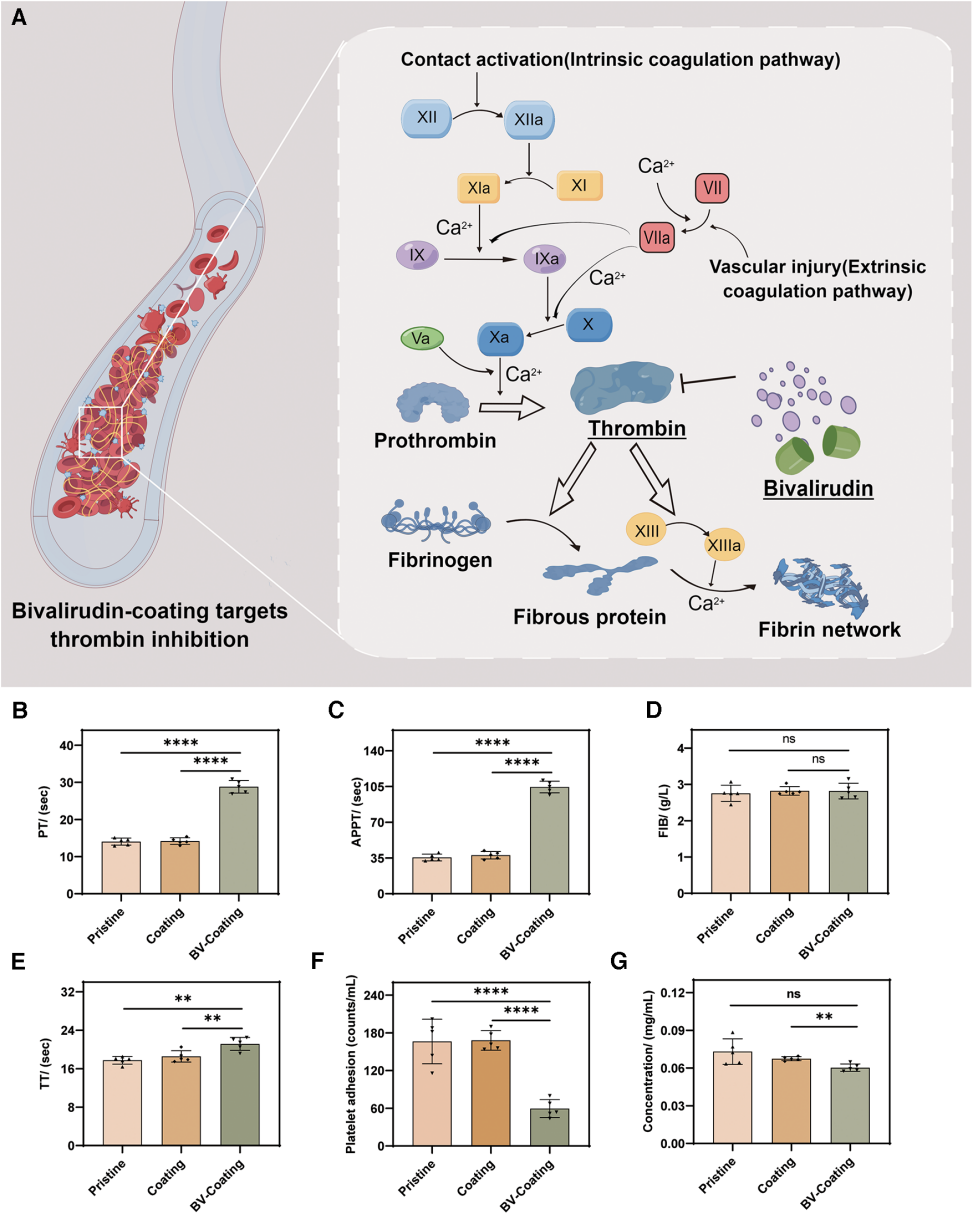
*In vitro* anticoagulant properties assay. (**A**) Schematic diagram of BV targeting thrombin; (**B**) PT activity; (**C**) APTT activity; (**D**) FIB activity; (**E**) TT activity; (**F**) Platelet adhesion assay; (**G**) Protein adsorption assay. (****P < 0.0001; **P < 0.01; ns, not significant).

Coagulation assays were selected to evaluate the antithrombotic properties of the three material groups. After incubation, the schematic diagram of PT, APTT, FIB, and TT expression levels in the blood samples from three material groups is in Figure 4A. As shown in Figures 4B–E, the FIB results showed no significant differences. However, in the APTT, TT, and PT assays, the BV-Coating group exhibited significantly prolonged clotting times compared to the other groups (*P *< 0.01). Thus, the BV-Coating GelMA hydrogel tube could significantly improve clotting phenomena.

### Platelet adhesion and protein adsorption without flowing *in vitro*

The adhesion of platelets and covalently cross-linked protein on the surface are the main factors of thrombosis (22). As shown in Figures 4F,G, the BV-Coating GelMA hydrogel tube showed a significant decrease in platelet adhesion and protein adsorption to the materials compared to the pristine and GelMA hydrogel tube (*P *< 0.01). This observation underscores the effectiveness of BV in mitigating platelet adhesion and protein adsorption when compared to both pristine PVC tubes and GelMA hydrogel coating tubes.

### Simulate the circulation *in vitro*

**Figure 5:**
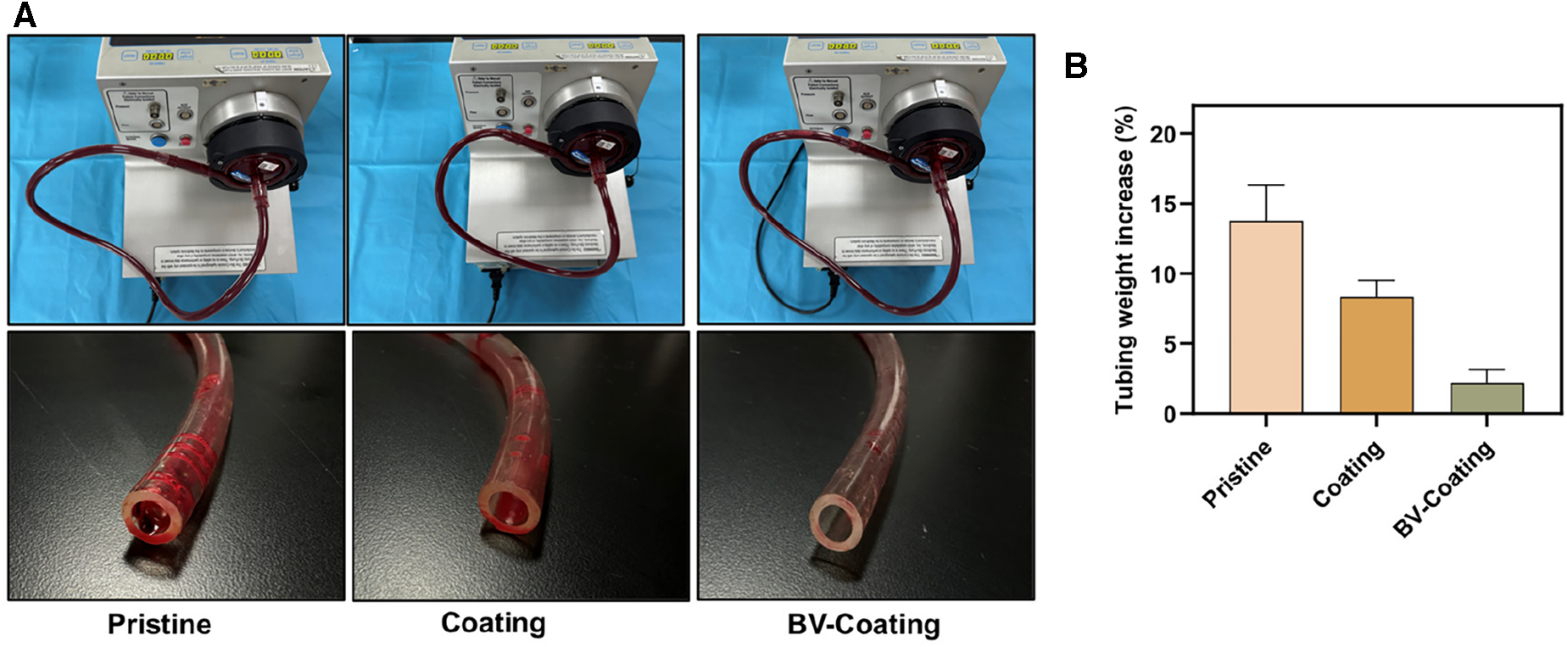
*In vitro* blood loop tests of dynamic blood. (**A**) Image of circulation condition and tubes after flow testing; (**B**) The occlusion rate of tubes after circulation of dynamic blood.

The ECMO machine centrifugal pump was utilized to simulate the circulation of whole blood in the ECMO system. As was shown in Figure 5A, compared with BV-Coating GelMA hydrogel tubes, the entry part of PVC and GelMA hydrogel coating tubes had more adhesive blood clots.

Following circulation, the pristine PVC and GelMA hydrogel coating tubes demonstrated weight increments of 13.8% and 8.3%, respectively, whereas the BV-Coating GelMA hydrogel tube experienced a mere 2.2% weight increase (Figure 5B). This observation suggested that the BV-Coating GelMA hydrogel tube could also exhibit effective anticoagulation properties during dynamic blood circulation.

### Antithrombogenicity assay *in vivo*

**Figure 6:**
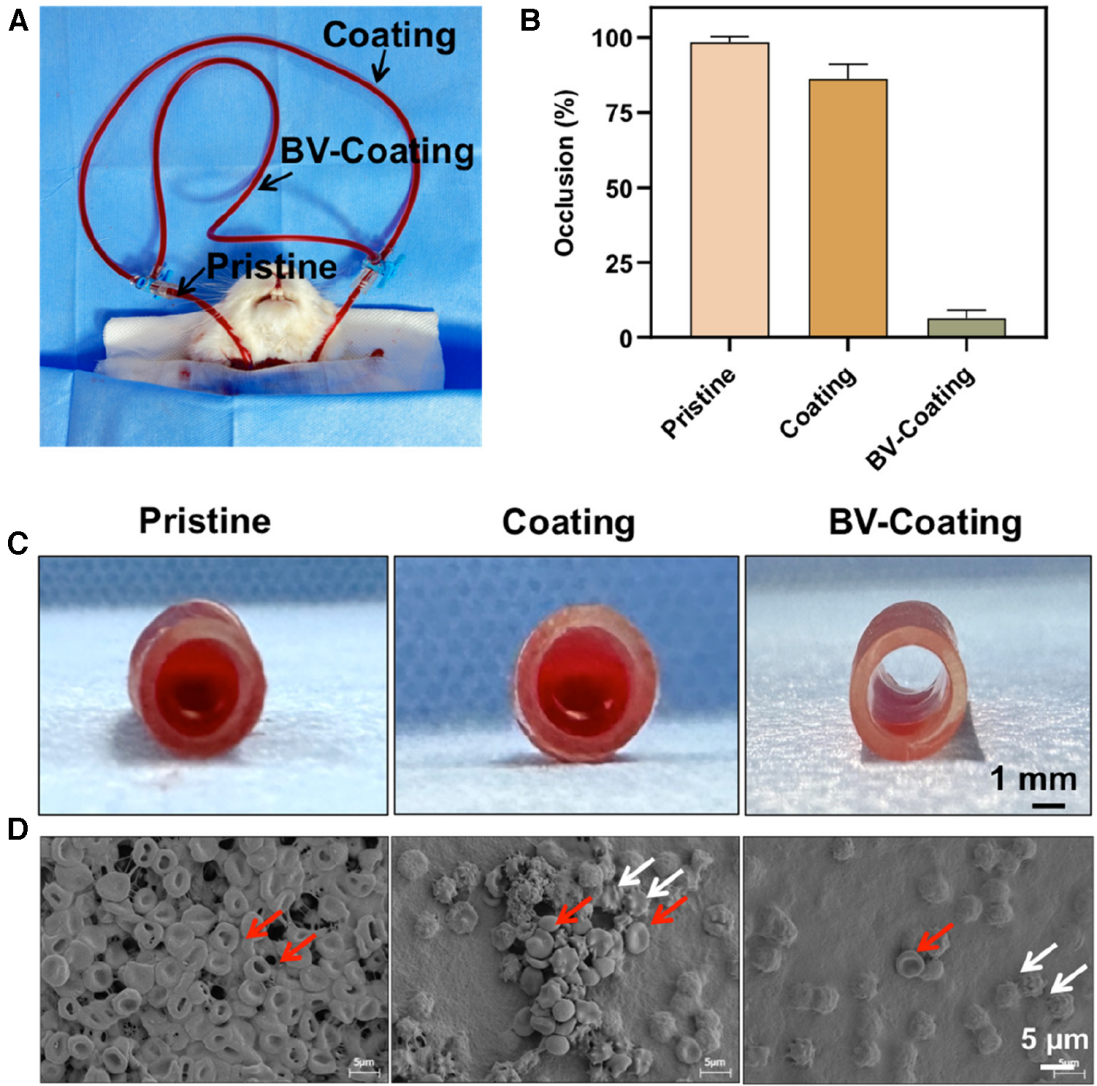
*In vivo* antithrombogenicity assay. (**A**) Schematic diagram of the arteriovenous shunt model; (**B**) Quantification of blood clotting adhesion to the tube walls after circulation; (**C**) The image of the pristine tube, GelMA-hydrogel coatings tube, and BV-Coating GelMA hydrogel tube after circulation. (**D**) Electron microscopy images of pristine tube, GelMA-hydrogel coatings tube, and BV-Coating GelMA hydrogel tube after circulation, red arrows indicate erythrocytes, white arrows indicate activated platelets.

In-vivo blood circulation model was chosen to further simulate the interaction between the materials and circulating whole blood. As illustrated in Figure 6A, GelMA hydrogel coating loaded or not loaded with BV was parallelly integrated into a pristine PVC tube and connected to the blood vessels of the rabbit carotid artery and jugular vein. After a 4-hour circulation, Severe thrombotic occlusion was observed in the pristine PVC tube (98.50% ± 1.73%), and a small amount of thrombosis was observed in the GelMA hydrogel coating tube (86.33% ± 4.77%). Interestingly, the BV-Coating GelMA hydrogel tube showed minimal clotting (6.37% ± 2.80%) (Figure 6B). Complete thrombotic occlusion was observed within the lumen of the pristine PVC tube, while the BV-Coating GelMA hydrogel tube maintained a high patency rate (Figure 6C). SEM was employed to scrutinize blood adhesion on the surface of the tube (Figure 6D). Multiple thrombins composed of platelets and erythrocytes were observed on the exposed surfaces of the PVC and GelMA hydrogel coating tube. However, the surface of the BV-Coating GelMA hydrogel tube remained remarkably pristine, with only a minimal number of adhered erythrocytes and platelets. The findings further affirmed that the BV-Coating GelMA hydrogel tube exerts a potent inhibitory effect on intravascular thrombus formation.

### Comparison of systemic anticoagulant assay *in vivo*

To further compare the anticoagulant effect of BV-Coating GelMA hydrogel on the systemic system with BV-iv, the results of clotting factors, PT, APTT, and bleeding time of BV-iv group and BV-coating group were compared *in vivo* blood circulation model.

After circulation, there was no apparent difference in clotting factors (II, V, and X) among the three groups (Supplementary Figure S4). However, compared with the pristine PVC group, PT, APTT, and bleeding time were significantly increased in PVC with the intravenous BV group (*P *< 0.01), but no significant change in the BV-Coating GelMA hydrogel group (Supplementary Figure S5). After completing the *in vivo* experiment, the Pristine tube group experienced severe thrombus occlusion, while the BV-GelMA hydrogel tube group and BV-iv group only had a small amount of thrombus (Supplementary Figure S6). The BV-GelMA hydrogel tube can achieve anticoagulant effects on local pipelines without affecting the overall coagulation level (Supplementary Figure S7).

## Discussion

Following the Extracorporeal Life Support Organization registry result, among 78,397 patients who received ECMO assistance from 1989 to 2016, only 58% were able to survive and be discharged (23). In addition to underlying conditions, adverse events related to biocompatibility, such as bleeding, thrombosis, and systemic inflammation, are also significant factors influencing prognosis. The surface of the ECMO tube is different from that of the human vascular endothelium. When it comes into contact with human blood, it triggers a strong immune response, which could cause serious complications (24). Thus, creating ECMO coating materials highly compatible with the body and have anticoagulant properties is essential to prevent these conditions.

The GelMA hydrogel coating that contains BV was successfully produced, which acts as a direct thrombin inhibitor by binding to the anion-binding exosite of both free and clot-bound catalytic sites of thrombin. Compared with heparin, BV has better safety, reliability, and cost-effectiveness (25). Importantly, the BV-Coating GelMA hydrogel tube did not significantly affect cell growth activity nor elicit hemolysis, complement activation, or coagulation reactions when in contact with blood. In addition, the BV-Coating GelMA hydrogel tube did not cause any harmful thrombus formation in the body.

In clinical practice, pre-primed ECMO tubes are typically used for up to 7 days (26). The release effect was calculated for seven days, and it was found that the rapid release period of BV occurs within the first three days, which is probably due to the significant concentration difference between the BV-Coating GelMA hydrogel tube and the PBS. Hydrogel has a good three-dimensional network structure, BV could not only adsorb on the surface of the gel but also enter the inside of the gel mesh and be wrapped in the gel mesh, forming a skeleton drug loading system. Therefore, both Higuchi and Ritger-Peppas models could describe the kinetic process of BV release in a BV-Coating GelMA hydrogel tube.

Activated complement components could facilitate leukocyte adhesion and activation on material surfaces, leading to localized and systemic inflammatory reactions (27). Under the action of complement 3 lyase (C3), C3 could undergo cleavage, resulting in the generation of C3a and C3b. Interestingly, compared to the coating and negative controls, the introduction of BV did not significantly increase the concentration of C3a, indicating that BV could not induce complement activation. Thomas et al. (28) also reported in their clinical trials that BV does not cause complement activation in human blood.

Apart from the complement, platelets in the blood can become more active during an ECMO treatment and release substances that cause inflammation (29). As a marker of platelet activation, β-TG is a specific protein released from platelets into the plasma in response to appropriate stimuli. BV-Coating GelMA hydrogel tube could not increase β-TG levels, indicating that it does not increase excess platelet activation.

Regarding anticoagulation, the BV-Coating GelMA hydrogel tube showed significantly prolonged PT and APTT compared to the pristine PVC tube and GelMA hydrogel coating tube, indicating the effective anticoagulant property of GelMA hydrogel delivering BV. The potential of BV coating GelMA hydrogel tube in improving the anticoagulation effect. The underlying mechanism lies in the ability of BV to inhibit thrombin, thereby blocking the coagulation pathway and extending the clotting time (Figure 4A). Similar findings have been reported by Yang et al. (30), further affirming the capacity of BV to prolong clotting time and enhance the blood compatibility of biomaterials.

Adhesion of platelets and protein adsorption plays an essential role during the coagulation process. The adhesion of platelets to the material surface directly contributes to thrombus formation. Once the biomaterial comes into contact with blood, platelets aggregate and form clots, and plasma proteins rapidly adsorb onto the surface. The adsorbed proteins could mediate platelet adhesion and aggregation, leading to the eventual formation of blood clots (31). Simultaneously, fibrinogen plays a crucial role in the process of thrombus formation. Under the action of thrombin, fibrinogen undergoes proteolytic cleavage, transforming into fibrin, which forms the intricate meshwork of the clot's foundation (32). BV-Coating GelMA hydrogel tube did not significantly increase protein and platelet adsorption compared to the GelMA hydrogel coating tube and pristine PVC tube.

Blood-contacting biomaterials, such as vascular patches and grafts, have been evaluated for their blood compatibility in rabbit models (33), which could appropriately simulate the real environment of the human during extracorporeal circulation. Compared with intravenous administration, BV-Coating of the GelMA hydrogel tube could not affect the overall coagulation status, avoiding the risk of systemic bleeding at the therapeutic level. However, the effect of local anticoagulation could be achieved through the elution of blood contact, which has been confirmed through a parallel rabbit circulation model (Figure 6).

There are still some issues that need further consideration and improvement regarding the standardization of characterization of anticoagulant coatings. Firstly, although the parallel rabbit circulation model avoids differences in individual coagulation levels among different animals, it inevitably leads to the problem of mutual influence between pipelines. Secondly, although thrombosis is minimal in the BV-Coating of the GelMA hydrogel group, there is a certain angle between the GelMA hydrogel coating pipeline and the direction of blood flow, which inevitably reduces the blood flow velocity in the pipeline and exacerbates the formation of thrombosis. Also, BV is administered intravenously. There are currently no pipeline coating products with BV, we are unable to detect the differences in drug release between Coating of the GelMA hydrogel and other coating methods at the same BV dose.

By releasing BV locally, the risk of complications of systemic anticoagulant therapy could be avoided while improving the availability of BV. A novel anticoagulant coating strategy has a good application prospect in medical PVC tubes.

## Conclusion

As an anticoagulant material, the application of heparin coating was limited by the potential risk of complications such as HIT and essential hemorrhage. Given its shorter drug metabolism and more precise mechanism of action, BV could hold more significant potential for application in tube coating technology. The BV-Coating of the GelMA hydrogel tube was successfully synthesized and substantiated its good biocompatibility and surface anticoagulation. Compared with intravenous injection, BV hydrogel coating does not significantly alter the overall coagulation level, which can prevent complications such as systemic bleeding while promoting the treatment process. The GelMA hydrogel coating technology represents a design strategy that mitigates the risk of thromboembolism associated with using blood-contacting medical devices.

## Data Availability

The original contributions presented in the study are included in the article/**Supplementary Material**, further inquiries can be directed to the corresponding authors.
