# Supplementary material for: Bivalirudin-hydrogel coatings of polyvinyl chloride on extracorporeal membrane oxygenation for anticoagulation
Source: Front Cardiovasc Med. 2023 Dec 15;10:1301507. doi: 10.3389/fcvm.2023.1301507 (PMC10754995; doi:10.3389/fcvm.2023.1301507)
Supplement: Supplementary file 1 [file Datasheet1.docx]

**Supplemental Figure1** The image of GelMA-hydrogel coatings tube.

**Supplemental Figure2**. Schematic illustration of polymer networks on various solid surfaces**.**

**Supplemental Figure3** Schematic illustration of the synthesis of GelMA hydrogel coating layer with BV and the anticoagulant effect of ECMO tube.

**Supplemental Figure4** Comparison of clotting factors in systemic anticoagulation assay.

**Supplemental Figure5** Comparison of PT/APTT in systemic anticoagulation assay.

**Supplemental Figure6** Comparison of bleeding time in systemic anticoagulation assay

**Supplemental Figure7** The image of pristine tube, GelMA-hydrogel coatings tube, and BV-Coating GelMA hydrogel tube after circulation. (A) Pristine tube; (B) GelMA-hydrogel coatings tube; (C) BV-Coating GelMA hydrogel tube.

**Supplemental Table 1:** The fitting equations and fitting parameters of the release model of BV in BV-coating.


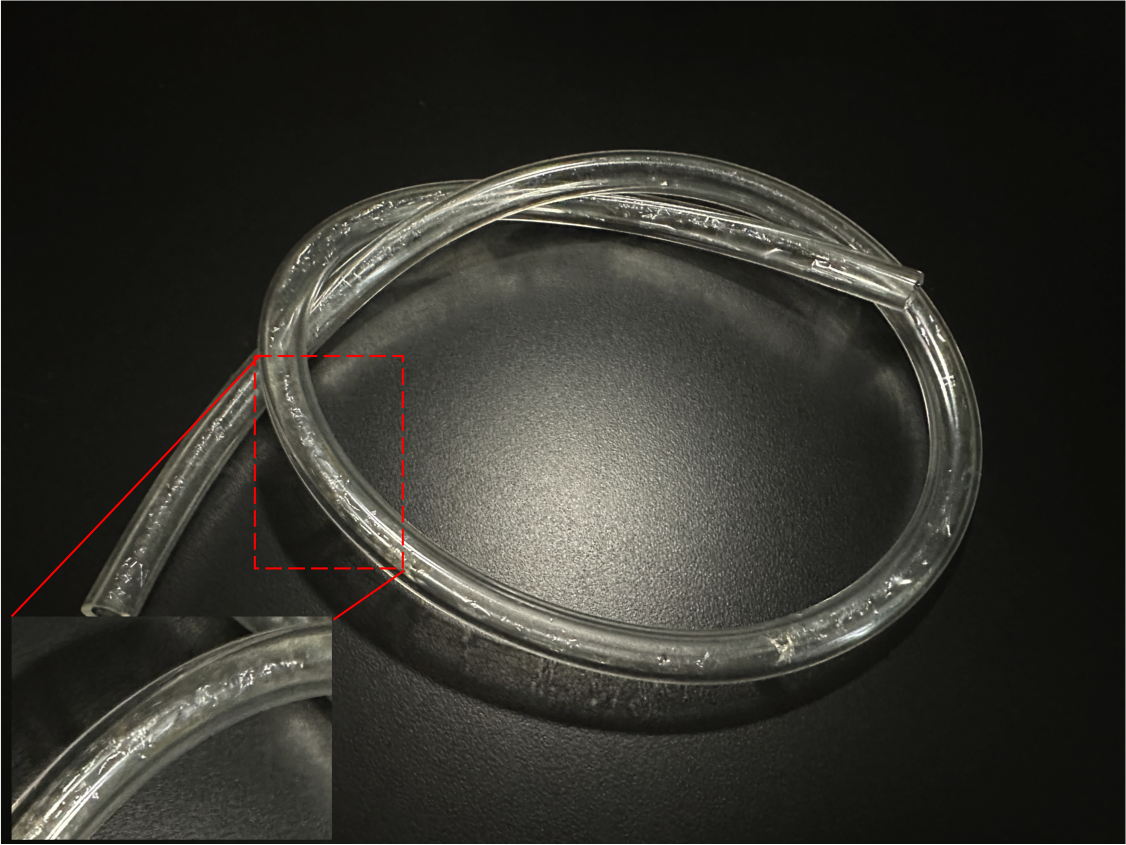


**Supplemental Figure1** The image of GelMA-hydrogel coatings tube.


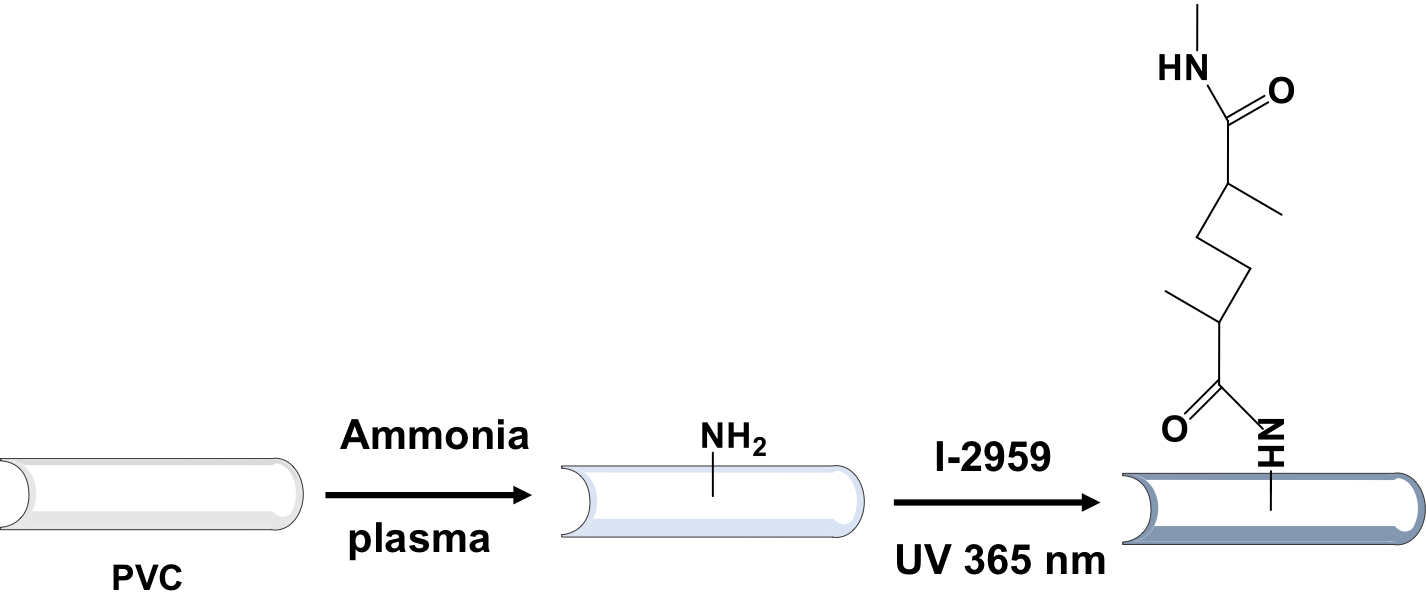


**Supplemental Figure2**. Schematic illustration of polymer networks on various solid surfaces**.**


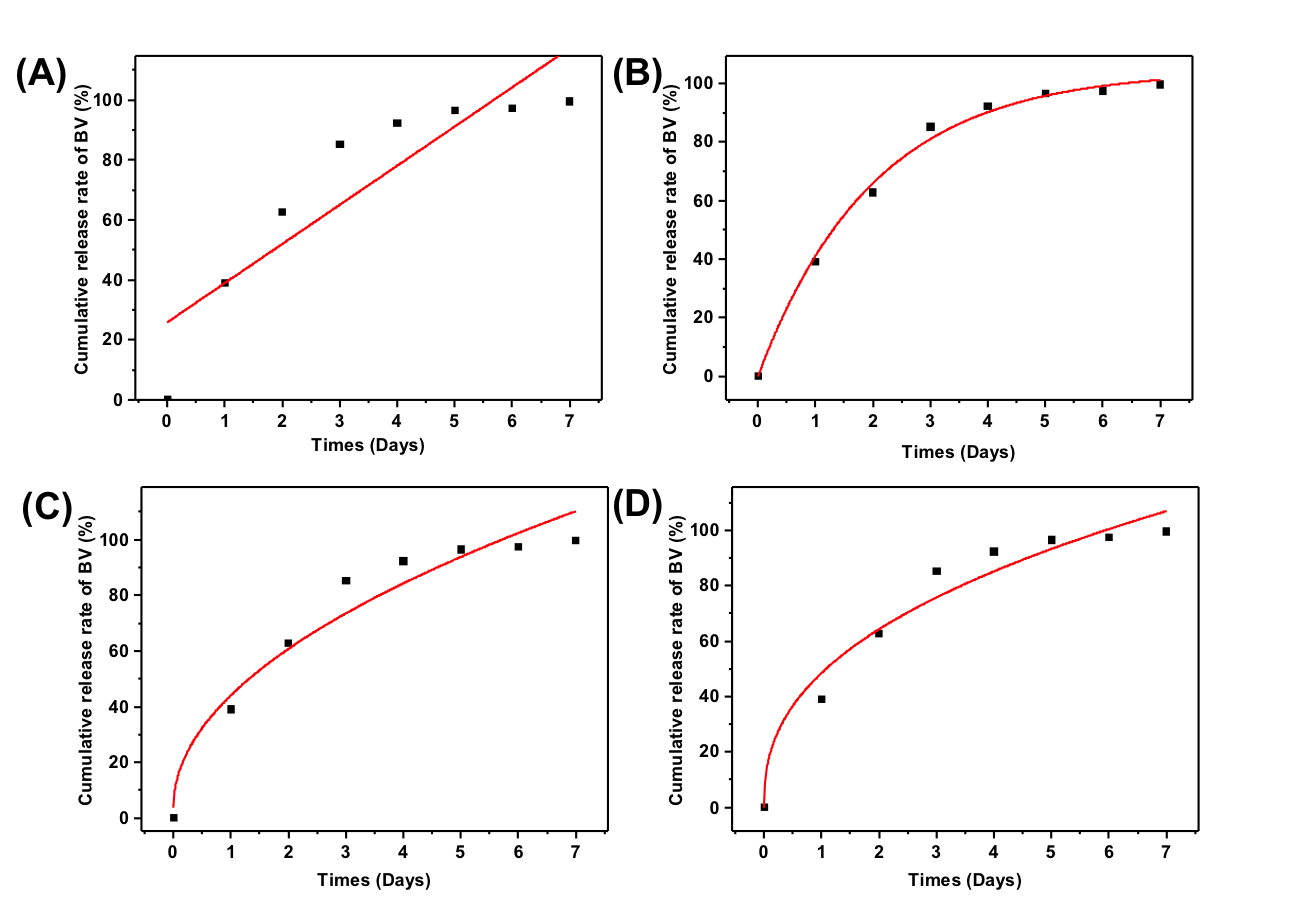


**Supplemental Figure3** Schematic illustration of the synthesis of GelMA hydrogel coating layer with BV and the anticoagulant effect of ECMO tube.

**
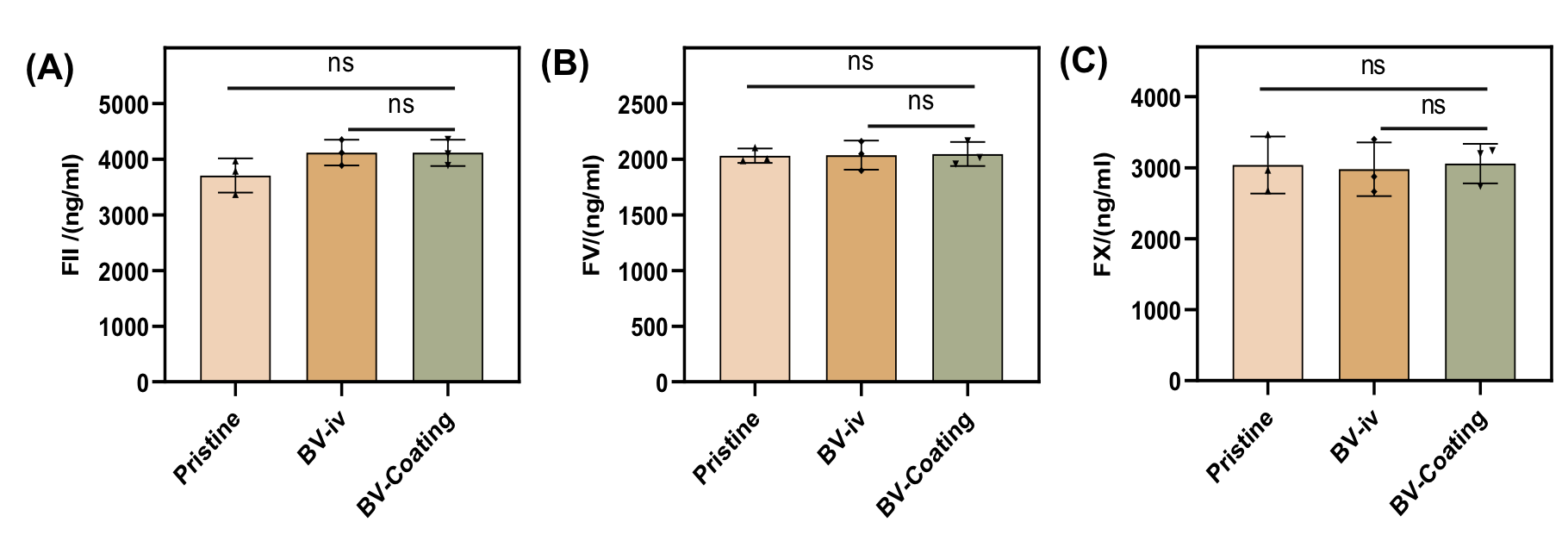
**

**Supplemental Figure4** Comparison of clotting factors in systemic anticoagulation assay. (A) Clotting factor II concentration; (B) Clotting factor V concentration; (C) Clotting factor X concentration. (ns, not significant).


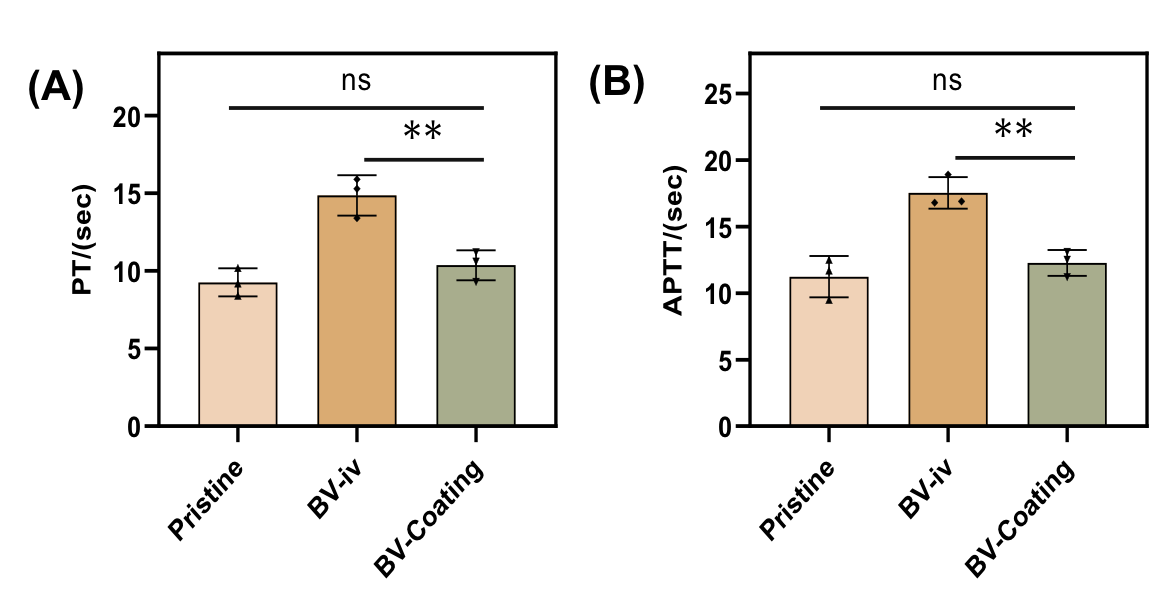


**Supplemental Figure5** Comparison of PT/APTT in systemic anticoagulation assay. (A) PT activity; (B) APTT activity; (***p* < 0.01; ns, not significant).


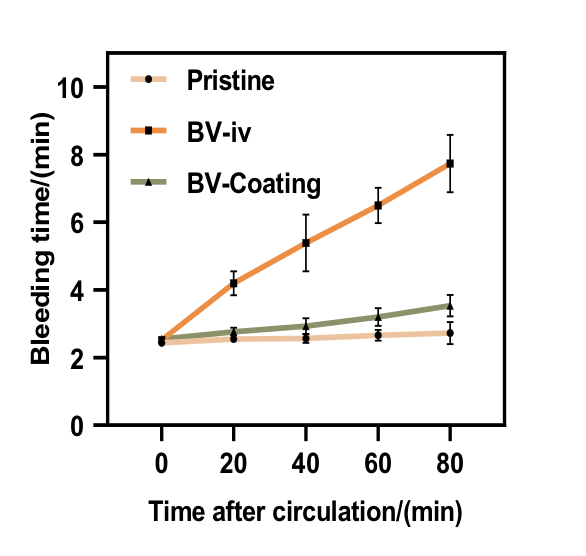


**Supplemental Figure6** Comparison of bleeding time in systemic anticoagulation assay. (***p* < 0.01; ns, not significant).


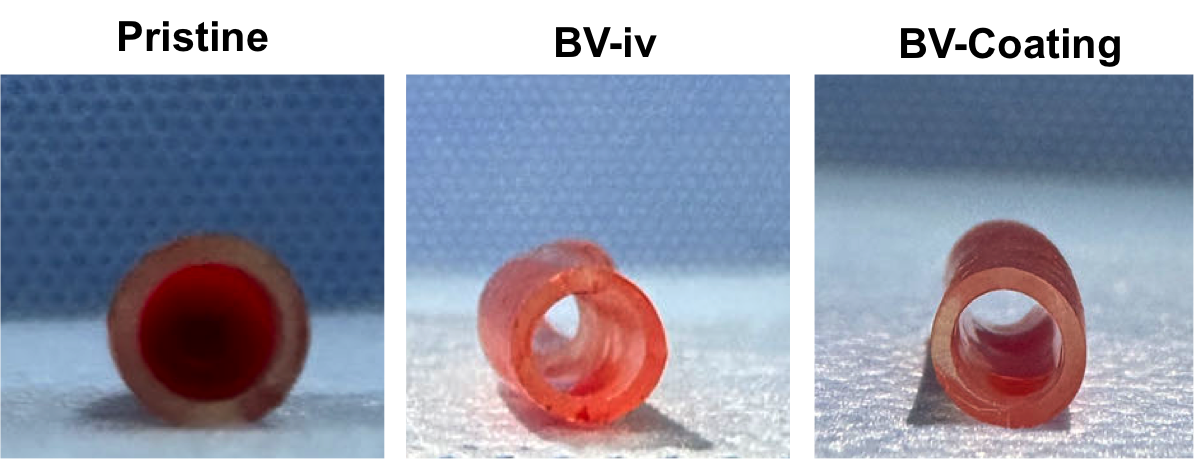


**Supplemental Figure7** The image of pristine tube, GelMA-hydrogel coatings tube, and BV-Coating GelMA hydrogel tube after circulation. (A) Pristine tube; (B) GelMA-hydrogel coatings tube; (C) BV-Coating GelMA hydrogel tube.

**Supplemental Table 1:** The fitting equations and fitting parameters of the release model of BV in BV-coating.

| Model | Equation | Adjusted-R^2^ |
| --- | --- | --- |
| zero-order release model | Q_t_=13.07t+25.88 | 0.76809 |
| first-order release model | Q_t_=104.33(1-e^-0.50t^) | 0.99461 |
| Higuchi release model, | Q_t_=40.21t^1/2^+3.870 | 0.94925 |
| Ritger-Peppas release model | Q_t_=48.45t^0.41^ | 0.96048 |
